# Construction and Experimental Validation of a Quantitative Kinetic Model of Nitric Oxide Stress in Enterohemorrhagic *Escherichia coli* O157:H7

**DOI:** 10.3390/bioengineering3010009

**Published:** 2016-02-06

**Authors:** Jonathan L. Robinson, Mark P. Brynildsen

**Affiliations:** Department of Chemical and Biological Engineering, Princeton University, Princeton, NJ 08544, USA; jlrtwo@princeton.edu

**Keywords:** nitric oxide, enterohemorrhagic *E. coli*, kinetic model, ensemble modeling, Hmp, NorV, microaerobic, anaerobic

## Abstract

Enterohemorrhagic *Escherichia coli* (EHEC) are responsible for large outbreaks of hemorrhagic colitis, which can progress to life-threatening hemolytic uremic syndrome (HUS) due to the release of Shiga-like toxins (Stx). The presence of a functional nitric oxide (NO·) reductase (NorV), which protects EHEC from NO· produced by immune cells, was previously found to correlate with high HUS incidence, and it was shown that NorV activity enabled prolonged EHEC survival and increased Stx production within macrophages. To enable quantitative study of EHEC NO· defenses and facilitate the development of NO·-potentiating therapeutics, we translated an existing kinetic model of the *E. coli* K-12 NO· response to an EHEC O157:H7 strain. To do this, we trained uncertain model parameters on measurements of [NO·] and [O_2_] in EHEC cultures, assessed parametric and prediction uncertainty with the use of a Markov chain Monte Carlo approach, and confirmed the predictive accuracy of the model with experimental data from genetic mutants lacking NorV or Hmp (NO· dioxygenase). Collectively, these results establish a methodology for the translation of quantitative models of NO· stress in model organisms to pathogenic sub-species, which is a critical step toward the application of these models for the study of infectious disease.

## 1. Introduction

Pathogenic *Escherichia coli* are responsible for a broad range of infections within humans depending on their pathotype, and these can generally be classified as enteric (diarrheagenic) or extraintestinal pathogenic *E. coli* (ExPEC) [1,2,3]. ExPEC can cause infections at most locations within the body (e.g., meningitis, pneumonia, sepsis, abdominal infection) [1], and the most common pathotype is uropathogenic *E. coli* (UPEC) [4], which are responsible for ~65%–75% of urinary tract infections (UTIs) [5]. Diarrheagenic *E. coli* are generally divided into six pathotypes; enterotoxigenic (ETEC), enteropathogenic (EPEC), enteroaggregative (EAEC), enteroinvasive (EIEC), diffusely adherent (DAEC), and enterohemorrhagic (EHEC). EHEC are potentially deadly, food-borne pathogens responsible for large outbreaks of hemorrhagic colitis (bloody diarrhea). These outbreaks often receive international attention, such as the 1996 outbreak in Japan that sickened over 8000 people [6], the 2006 spinach contamination in California affecting 205 individuals [7,8], and more recently the 2011 outbreak in Germany where ~4000 cases led to the highest incidence of hemolytic uremic syndrome (HUS) on record [9,10].

HUS can occur in up to 20%–25% of patients, and is the leading cause of death from EHEC [3,11,12]. The condition is caused by EHEC Shiga-like toxins (Stx) that are harbored on prophage and released during the phage lytic cycle in response to DNA damage [13]. When released, these toxins proceed to attack renal tissue where they inhibit translation through disruption of the 60S ribosomal subunit, leading to kidney failure [3,7,14,15,16]. Unfortunately, antibiotics are not recommended for treatment of EHEC infections for several reasons: (1) fluoroquinolones can stimulate prophage induction and Stx release; (2) β-lactams lyse EHEC to increase Stx exposure; and (3) destruction of the gut microbiome can enhance Stx absorption [3,11,17,18]. Furthermore, effective vaccines have yet to be developed, due to the challenges associated with a lack of human disease symptoms in EHEC-infected murine models, and Stx neutralizers have yet to show improved patient outcomes [3,11,19]. For these reasons, treatment of EHEC is largely restricted to supportive care [3], and the frequent outbreaks serve as important reminders of how helpless we are in our fight against these pathogens [11].

Kulasekara and colleagues conducted a genomic analysis of 100 different EHEC isolates, and found that the presence of a functional NO· reductase (NorV) correlated with an increased incidence of HUS [7]. EHEC strains possessing a functional NorV enzyme were associated with HUS incidences of up to 25%, whereas those with the inactive form of the enzyme (possessing a 204 nt in-frame deletion in the *norV* gene) exhibited fewer cases (up to a 10-fold reduction) of HUS [7,10]. Recent outbreaks have supported this association, such as the German outbreak in 2011, where the causative O104:H4 strain possessed a functional NorV [10]. The greater severity of infections caused by functional NorV-bearing EHEC isolates has been attributed to the protection NorV provides from the nitrosative stress exerted by immune cells, such as macrophages [20]. NorV is the main anaerobic NO· detoxification enzyme in *E. coli* [21,22,23,24], and NorV-proficient EHEC have been shown to exhibit reduced NO· levels, prolonged survival, and increased Stx production within macrophages compared to their NorV-deficient counterparts [20].

Under aerobic conditions, the major NO· detoxification system in *E. coli* is NO· dioxygenase (Hmp) [25,26,27]. Hmp has been identified as a virulence factor in many pathogens [28], including UPEC [29], *Salmonella enterica* serovar Typhimurium [30,31], *Yersinia pestis* [32], *Staphylococcus aureus* [33], and *Vibrio cholerae* [34]. Interestingly, a study by Vareille and colleagues found that NO· inhibits Stx production and release from EHEC under oxygenated conditions [35]. Likewise, Branchu and colleagues demonstrated that NO· inhibits the expression of many genes of the EHEC enterocyte effacement (LEE) pathogenicity island under aerobic conditions [36]. Since the vast majority of NO· in *E. coli* cultures is detoxified by Hmp under such conditions [23,25,27], it is tempting to postulate that EHEC can use Hmp to promote expression of its primary virulence factors under NO· stress in the presence of O_2_.

Since inhibitors of NorV or Hmp would have the potential to reduce kidney failure and death from EHEC, and humans do not possess a homologue to either protein (Section 2.10), they represent attractive targets for the development of therapies for EHEC infections. Furthermore, inhibitors specific to NO· detoxification enzymes should minimally impact the normal microbiome because NO· defenses are not essential functions for bacterial growth under normal conditions. Unfortunately, known chemical inhibitors of NO· reductases or dioxygenases (e.g., carbon monoxide or cyanide [37,38]) are toxic to humans, and have a greatly weakened effect on the flavodiiron active site used by the *E. coli* NorV [22,23]. Alternative targets in the NO· defense network of EHEC must therefore be identified; however, the broad reactivity of NO· and its reaction products give rise to a large, complex, and interconnected reaction network that includes biological effects ranging from iron-sulfur cluster destruction to inhibition of respiration and DNA damage [27,28,39,40,41]. The biological outcome of NO· exposure is dictated by a complex kinetic competition within this network, which necessitates the use of computational models for accurate interpretation, understanding, and analysis [27,28]. Such models can quantify the impact of different perturbations on the NO· response, and aid in identifying the underlying mechanisms [27,28,42,43]. Because previous models of NO· stress were developed for a non-pathogenic model organism (*E. coli* K-12), we sought to translate this approach and demonstrate its performance in the clinically-relevant pathogen, EHEC.

Here, we have constructed a quantitative kinetic model of the NO· stress response network in EHEC, which is composed of a system of differential mass balances that was translated from *E. coli* K-12 through a process involving literature and database examination, BLAST comparison of protein sequences, and relaxation of uncertain parameters. The model was trained on experimental measurements of [O_2_] and [NO·] from EHEC cultures and an MCMC algorithm was employed to populate an ensemble of viable models that could be used to assess parametric and prediction uncertainty. Given the low O_2_ tension typically associated with EHEC infection sites, experiments were performed under microaerobic (50 μM O_2_) and anaerobic (0 μM O_2_) conditions. The trained ensemble of models was able to quantitatively capture [NO·] dynamics in EHEC cultures treated with an NO·-releasing chemical, DPTA NONOate, under both O_2_ conditions. Furthermore, the ensemble was used to make forward predictions of [NO·] dynamics in cultures of EHEC mutants lacking either of the two major NO· detoxification enzymes, NorV and Hmp. The corresponding experiments were performed, and measurements exhibited excellent agreement with predictions. These results demonstrate that quantitative kinetic modeling of NO· stress can be extended to clinically-relevant strains under physiological O_2_ environments, which will facilitate deeper understanding of EHEC NO· defenses and could foster the development of alternative therapeutics for EHEC infections.

## 2. Materials and Methods

### 2.1. Chemicals and Growth Media

Cells were grown in Luria-Bertani (LB) Broth (BD Difco), or MOPS minimal media (Teknova) with 10 mM glucose. NO· was delivered to the cultures using DPTA NONOate ((Z)-1-[*N*-(3-aminopropyl)-*N*-(3-ammoniopropyl)amino]diazen-1-ium-1,2-diolate) (Cayman Chemical, Ann Arbor, MI, USA), which spontaneously dissociates with a half-life of ~2.5 h at 37 °C and pH 7.4 to release 2 equivalents of NO· per parent compound. For plasmid retention, all growth media contained 30 μg/mL kanamycin (Fisher Scientific) under oxygenated conditions, or 100 μg/mL kanamycin for anaerobic conditions (due to the reduced activity of kanamycin in the absence of respiration).

### 2.2. Bacterial Strains

Strains used in this study (Table 1) were derived from enterohemorrhagic *E. coli* O157:H7 TUV93-0 [44]. This strain natively possesses a 204 nt in-frame deletion within the *norV* gene that renders the protein inactive. To introduce a functional *norV*, TUV93-0 was transformed with a plasmid harboring the intact gene and its promoter (Section 2.3), yielding the TUV93-0 *hmp*^+^/*norV*^+^ strain. The *norV*-null strain used here, TUV *hmp*^+^/*norV*^−^, refers to the native TUV93-0 transformed with an empty vector (pUA66 [45]), which was included to impart a similar metabolic burden to that in the *norV*^+^ strain. The TUV *hmp*^−^/*norV*^+^ strain was generated by replacing *hmp* on the TUV93-0 chromosome with a kan^R^ cassette using the lambda Red recombinase system [46], where the kan^R^ cassette DNA was amplified from the purified genomic DNA of the Δ*hmp*::kan^R^ mutant in the Keio Collection [47] using primers 5′-TGAGATACATCAATTAAGATGCAAAA-3′ (forward) and 5′-AAGGGTTGCCGGATGTTT-3′ (reverse). The mutant was cured of the kan^R^ marker using FLP recombinase encoded on the pCP20 plasmid [46], and confirmed via cPCR with two sets of primers: 5′-CCGAATCATTGTGCGATAACA-3′ with 5′-GCAAAATCGGTGACGGTAAA-3′ to check for the scar sequence, and 5′-TCCCTTTACTGGTGGAAACG-3′ with 5′-CACGCCCAGATCCACTAACT-3′ to confirm absence of *hmp* in the genome. The cured TUV Δ*hmp* mutant was then transformed with the *norV* complementation plasmid to restore NorV functionality, yielding the TUV *hmp*^−^/*norV*^+^ strain.

**Table 1:** *E. coli* strains used in the present study.

Strain	Genotype	Reference
TUV93-0	EHEC O157:H7 EDL933 Stx^−^	Leong, J.M. [44]
TUV *hmp*^+^/*norV*^+^	TUV93-0 + pUA66-P*_norV_*-*norV*	This work
TUV *hmp*^+^/*norV*^−^	TUV93-0 + pUA66	This work
TUV *hmp*^−^/*norV*^+^	TUV93-0 Δ*hmp* + pUA66-P*_norV_*-*norV*	This work

### 2.3. Plasmid Construction

The *norV* complementation plasmid (pUA66-P*_norV_*-*norV*) consisted of an SC101 origin of replication (low copy), a kan^R^ resistance marker, and the *norV* gene with 188 nt of its 5′ UTR (P*_norV_*), which was cloned from purified *E. coli* K-12 MG1655 genomic DNA. Only 188 nt of the 5′ UTR of *norV* was incorporated to avoid inclusion of the *norR* start codon, which begins 189 nt upstream of the *norV* coding sequence (on the complement DNA strand), and this 188 nt sequence is identical in *E. coli* K-12 and EHEC except for two single-nucleotide mutations. Briefly, the genomic DNA of an overnight culture of WT MG1655 grown in LB media was purified using the DNeasy Blood & Tissue Kit (Qiagen), following the manufacturer’s instructions for Gram-negative bacteria. The P*_norV_*-*norV* DNA fragment was amplified from the purified genomic DNA using Phusion^®^ High-Fidelity DNA Polymerase (New England Biolabs, Ipswich, MA, USA; NEB) with primers 5′-GCGCATCTCGAGTACGATCTTTGCCTCACTGTCAATTT-3′ (forward) and 5′-GCGCGGTCTAGATCATTTTGCCTCCGATG-3′ (reverse), which possessed XhoI and XbaI restriction enzyme (RE) cut sites, respectively. The P*_norV_*-*norV* amplicon was gel-purified with the QIAquick Gel Extraction Kit (Qiagen), RE digested with XhoI and XbaI (NEB), and PCR-purified (Qiagen). Meanwhile, purified pUA66 plasmid (possessing the SC101 origin and kan^R^ cassette) [45] was RE digested with XhoI and XbaI (NEB), PCR-purified, and treated with Antarctic Phosphatase (NEB) to prevent self-ligation of the linearized plasmid backbone. After Antarctic Phosphatase treatment, the plasmid backbone was PCR-purified. The P*_norV_*-*norV* DNA fragment and linearized plasmid backbone were ligated using the Quick Ligation™ Kit (NEB), and transformed into XL1-Blue competent cells (Zymo Research, Irvine, CA, USA), which were immediately plated onto LB-agar plates containing 50 μg/mL kanamycin. Colonies were selected and grown overnight in LB with 30 μg/mL kanamycin, and the plasmid was purified (QIAprep Spin Miniprep Kit, Qiagen). The plasmid sequence was confirmed with PCR and sequencing of the P*_norV_*-*norV* region of the plasmid (Genewiz).

### 2.4. Glovebox Setup and Operation

In order to perform measurements under microaerobic and anaerobic conditions, experiments were conducted in a Coy hypoxic chamber with an anaerobic upgrade. For microaerobic conditions, the atmosphere was maintained at 5.0% O_2_ *v*/*v* (corresponding to 50 μM dissolved O_2_ in MOPS minimal media at 37 °C), with 0.2% *v*/*v* CO_2_, and the balance N_2_. To achieve anaerobic conditions, the atmosphere contained 2% H_2_ to scavenge trace O_2_ via reduction to H_2_O on a palladium catalyst, as well as 0.2% CO_2_, and the balance N_2_.

### 2.5. Bioreactor Apparatus

A bioreactor was operated in the glovebox to facilitate NO· measurements in a culture exposed to environments with controlled O_2_ concentrations. The reactor consisted of a 50 mL conical tube with 10 mL of MOPS + 10 mM glucose media, open to the glovebox environment, and stirred constantly with a 0.5 inch magnetic stir bar. The conical tube was suspended in a magnetically stirred beaker of water maintained at 37 °C with a stirring hotplate.

### 2.6. NO· Treatment Assay

One mL of LB media with 10 mM glucose was inoculated with a small scrape of *E. coli* cells from a −80 °C frozen stock, and grown at 37 °C and 250 r.p.m. under aerobic conditions for 4 h. A new test tube of 1 mL fresh LB + 10 mM glucose was inoculated with 10 μL of the pregrowth, and placed in the glovebox to grow overnight (16 h) at 37 °C and 200 r.p.m. under O_2_ environments of 50 μM (microaerobic) or 0 μM (anaerobic). A 250 mL baffled shake flask containing 20 mL of fresh MOPS minimal media with 10 mM glucose, which had been equilibrated with the glovebox atmosphere overnight, was inoculated with the overnight culture to an OD_600_ of 0.01, and grown at 37 °C and 200 r.p.m. Upon reaching an OD_600_ of 0.2, the flask culture was used to inoculate the bioreactor (containing 10 mL of MOPS + 10 mM glucose) to an OD_600_ of 0.05, which was immediately treated with 50 μM DPTA NONOate. The concentration of NO· was monitored continuously for 1 h following treatment using an ISO-NOP electrode (World Precision Instruments).

### 2.7. Respiration (O_2_ Consumption) Assay

To train the respiratory module of the model, TUV *hmp*^+^/*norV*^+^ was grown identically as described for the NO· treatment assays (Section 2.6), except it was not treated with DPTA NONOate following inoculation into the bioreactor. Instead, dissolved [O_2_] in the culture was continuously monitored (1 read/sec) for up to 10 min post-inoculation, using a fiber-optic O_2_ sensor (FireStingO_2_ robust miniprobe; PyroScience).

### 2.8. Model Simulation

The kinetic model was constructed as described previously [27], and adapted for EHEC physiology using the process described in Section 3.1. Briefly, the model is composed of a set of ordinary differential equations (ODEs) describing the change in biochemical species concentrations over time, as a function of the associated reaction rates. Expressed in matrix form, the governing set of equations is written:

(1) $$\frac{dC}{dt}=S\cdot r$$

where ***C*** is a vector of biochemical species concentrations, ***S*** is the reaction stoichiometry matrix, and ***r*** is a vector of reaction rates. Reaction rates are a function of associated species concentrations and kinetic parameter values. The reactions and species were partitioned into intracellular and extracellular compartments to enable experimental parameterization and validation of model predictions [27,48], where NO· and O_2_ were assumed to diffuse rapidly across the membrane [49,50]. For further details on model compartmentalization, see [48]. Simulations were run in MATLAB (The MathWorks, Inc.) using the *ode15s* function to numerically integrate the system of ODEs, and solve for biochemical species concentrations as a function of time. The model is available for download on our research group website (https://www.princeton.edu/cbe/people/faculty/brynildsen/group/software/).

### 2.9. Parameter Optimization

Model parameters were optimized using a two-stage process. The first stage employed the MATLAB *lsqcurvefit* function, whereby parameter values were optimized such that the variance (σ^2^)-normalized sum of the squared residuals (SSR) between the measured (*y_meas_*) and simulated (*y_sim_*) data (e.g., NO· or O_2_ concentrations) was minimized:

(2) $$SSR=\sum_{i=1}^{n}\frac{{\left(y_{i,meas}-y_{i,sim}\right)}^{2}}{\sigma_{i,meas}^{2}}$$

The non-convex nature of the optimization problem yields local minima, which are dependent on the initial parameter values. To improve coverage of the solution space, least-squares minimizations were repeated 1000 times, each initiated with random parameter values drawn from a uniform distribution that spanned the permitted bounds.

The second stage of parameter optimization involved a Markov chain Monte Carlo (MCMC) method [51], whereby a random walk through parameter space was performed, starting from the best-fit (lowest SSR) parameter set obtained from the previous nonlinear least squares minimization. Relative quality of fit was quantified by the evidence ratio (ER), which was determined from the Akaike Information Criteria (AIC) corrected for small sample sizes [52,53], as described previously [48]. Briefly, the AIC was calculated for each parameter set using the following formula [54]:

(3) $${\text{AIC}}_{i}=n\ln\left(\frac{{\text{SSR}}_{i}}{n}\right)+2k+\frac{2k\left(k+1\right)}{n-k-1}$$

where *n* represents the number of data points and *k* is the number of fit parameters plus one (to account for the SSR estimation) [54]. The weight of evidence (*w*) of each parameter set in a collection of *P* parameter sets was calculated from the AIC values [54]:

(4) $$w_{i}=\frac{\exp(-\Delta_{i}/2)}{\sum_{i=1}^{P}\exp(-\Delta_{i}/2)},\;\Delta_{i}={\text{AIC}}_{i}-\min\left(\text{AIC}\right)$$

From the Akaike weights, the evidence ratios (ER) of each parameter set, which represents the likelihood of each parameter set relative to the best-fit parameter set, were calculated [55]:

(5) $${\text{ER}}_{i}=\frac{w_{\text{best}}}{w_{i}}$$

where *w*_best_ is the Akaike weight of the parameter set with the lowest AIC value (best fit). Parameter sets exhibiting an ER > 10 (*i.e.*, less than 10% as likely as the best-fit parameter set) were discarded. If the MCMC process improved the AIC such that the ER of the initial parameter set was greater than 10, the walk was repeated, using the new best-fit parameter set as the new initial set.

In addition to identifying more optimal parameter sets, the MCMC method revealed information on the flexibility, or confidence of parameter values. Confidence intervals (CIs) for parameters were calculated as the range of parameter values among all parameter sets with an ER < 10. Parameters with relatively narrow CIs were more informed by the optimization than those with a relatively wide CI.

### 2.10. BLAST Comparison of Proteins

Similarities of amino acid sequences between *E. coli* K-12 MG1655 and *E. coli* O157:H7 EDL933 proteins were determined via BLAST on the BioCyc Database [56], where MG1655 amino acid sequences were queried in the EDL933 genome with an expectation value threshold of 10, no filtering of query sequence for low-complexity regions, and the default BLOSUM62 substitution matrix.

For the searches of a Hmp or NorV homologue in humans, a BLAST analysis was conducted on the NCBI Database, querying for the *E. coli* EDL933 Hmp or *E. coli* MG1655 NorV amino acid sequence (MG1655 was used for NorV since the sequence is mutated in EDL933) in the *Homo sapiens* genome (Annotation Release 107) using default algorithm parameters (expectation threshold of 10, BLOSUM62 matrix).

## 3. Results

### 3.1. Model Translation from E. coli K-12 to Enterohemorrhagic E. coli O157:H7

**Figure 1:**
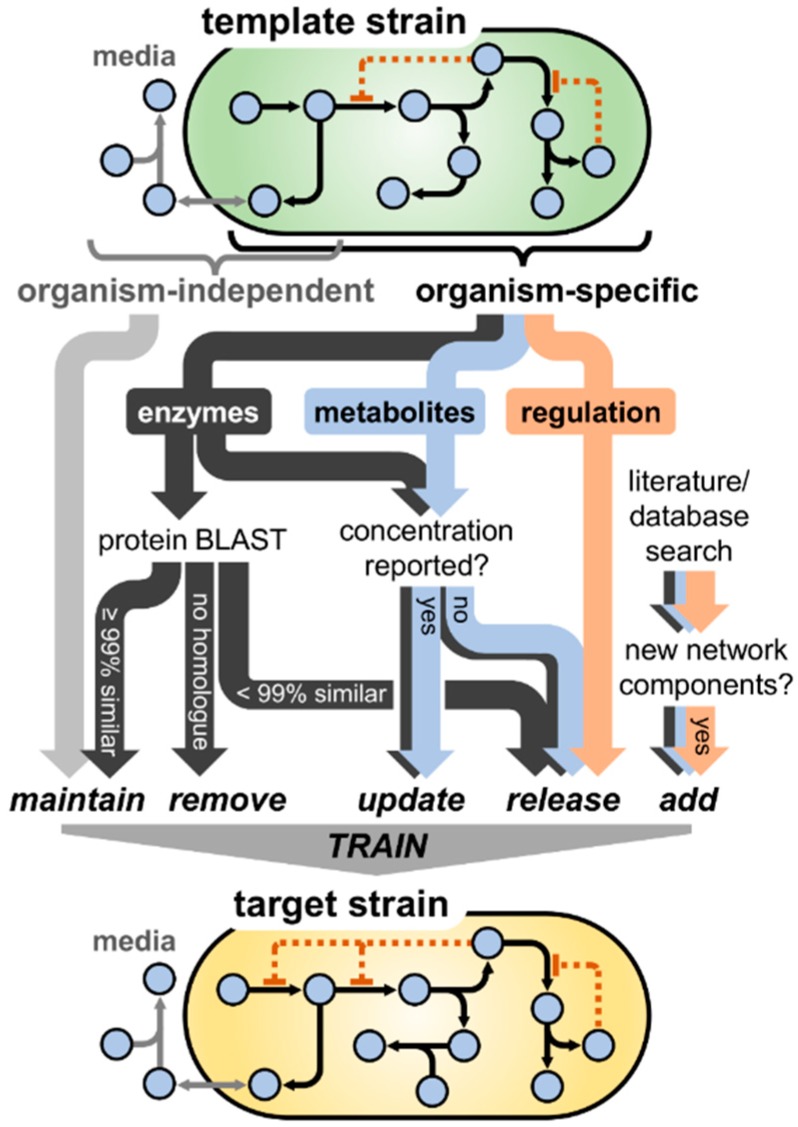
Schematic of the process of translating the NO· stress model from one bacterial strain (“template strain”) to another (“target strain”). Beginning with the original model constructed for the template strain, model components were categorized as organism-independent (e.g., NO· exchange with the gas phase) or organism-dependent (e.g., NO· reductase activity). Although the extracellular (media) compartment contains a high frequency of organism-independent components, many are also present intracellularly (e.g., NO· autoxidation). Parameters governing organism-independent processes were maintained at the same value, whereas those dependent on the species were handled differently depending on whether they governed enzyme activity, metabolite/biomolecule concentration, or transcriptional regulation. A BLAST analysis was conducted for each enzyme in the model, where parameters associated with enzymes exhibiting ≥99% amino acid similarity between the template and target strain were maintained at their original value, <99% similarity were released (allowed to vary) for subsequent training, and enzymes with no homologue in the target strain were removed entirely. If metabolite or enzyme concentrations were reported in the literature for the target strain, their values were updated in the model; however, if the concentrations had not been measured previously, they were released to be estimated during the training process. Given the relative complexity and large number of factors influencing the dynamics of transcriptional regulation, parameters governing these processes were released during model training. Finally, any additional network components (enzymes, metabolites, regulatory interactions) found in the literature to be involved in the NO· stress network of the target organism that were not present in the original construction were added to the model. Upon finalizing the model structure, the unknown/released parameters were trained (optimized) on experimental measurements with the target organism (e.g., NO· detoxification and respiratory O_2_ consumption).

To facilitate quantitative investigations of the EHEC NO· defense network, we constructed a kinetic model comprised of the relevant reactions and biomolecules involved in NO· stress. Beginning with a previously constructed model of the NO· response network of non-pathogenic *E. coli* (K-12 MG1655), the model was translated to represent EHEC physiology, accounting for potential and known differences in elements such as transcriptional regulation, metabolite concentrations, and enzyme kinetics. A schematic summarizing the model translation procedure is presented in Figure 1.

Sparse data existed in the literature on EHEC enzyme kinetics at the time of the present study, preventing the use of literature data to inform model parameters. We therefore performed a BLAST analysis to compare protein sequences between EDL933 (the parent of TUV93-0) and MG1655 for all enzymes in the model, with the assumption that enzymes with sufficiently similar amino acid sequences (≥99% match) would exhibit similar kinetics. Of the 20 enzymes (42 subunit proteins) present in the model, only 4 (5 subunits) exhibited amino acid sequences with less than 99% similarity between EDL933 and MG1655 (Table S1). These consisted of enzymes involved in DNA base excision repair (XthA and AlkA, 98.1% and 97.2% similar to that of MG1655, respectively), a subunit of NADH dehydrogenase I (NuoN, 87.2% similar), and NO· reductase NorVW (85.4% and 97.9% similarity for NorV and NorW, respectively). The difference in NorV for EDL933 has been noted previously, where this and some other EHEC strains possess a *norV* sequence with a 204 nt in-frame deletion that renders the protein inactive (commonly referred to as *norVs*) [7,20]. To enable analysis of both Hmp and NorV activity in the present study, a functional NorV was introduced into EHEC on a plasmid (Section 2.2 and Section 2.3).

Given the potential differences in metabolite concentrations and transcriptional regulation between the two *E. coli* strains, we relaxed all regulatory parameters and species concentrations, allowing them to vary within one order of magnitude of the MG1655 value when training on experimental measurements.

### 3.2. Model Parameter Training and Sensitivity Analysis

Extracellular parameters specific to the experimental apparatus (rate of DPTA NONOate dissociation, *k*_NONOate_, autoxidation rate of NO·, *k*_NO·-O2_, and the NO· volumetric mass transfer coefficient, *k*_L_*a*_NO·_) were trained on [NO·] measured in cell-free MOPS minimal media containing 10 mM glucose following treatment with 50 μM DPTA NONOate in an environment of 50 μM O_2_ (Figure S1 and Table S2). DPTA NONOate is an NO·-releasing chemical that dissociates with a half-life of approximately 2.5 h (at 37 °C, pH 7.4) to release two moles of NO· per mole of parent compound. Given the modest decrease in pH of anaerobically grown EHEC cultures (pH = 7.2 instead of 7.4), the DPTA release rate (k_NONOate_) was trained on cell-free MOPS media treated with 50 μM DPTA under anaerobic conditions, with the pH adjusted to 7.2 with HCl, while the other two extracellular parameters (*k*_NO·-O2_ and *k*_L_*a*_NO·_) were held constant. The volumetric mass transfer coefficient governing the exchange of dissolved O_2_ with the gas phase (*k*_L_*a*_O2_) was determined by measuring dissolved [O_2_] in cell-free media following degassing with N_2_. The data were plotted as ln([O_2_]_sat_—[O_2_]) *vs.* time, and a line was fit to the points, where the negative of the slope corresponded to the *k*_L_*a*_O2_ (1.25 × 10^−3^·s^−1^). After obtaining the extracellular parameter values, parameters governing the respiratory module (cytochrome ubiquinol oxidases and NADH reductases) were trained on O_2_ measurements in a culture of EHEC inoculated into the bioreactor to an OD_600_ of 0.05 (Figure S2 and Table S3).

**Figure 2:**
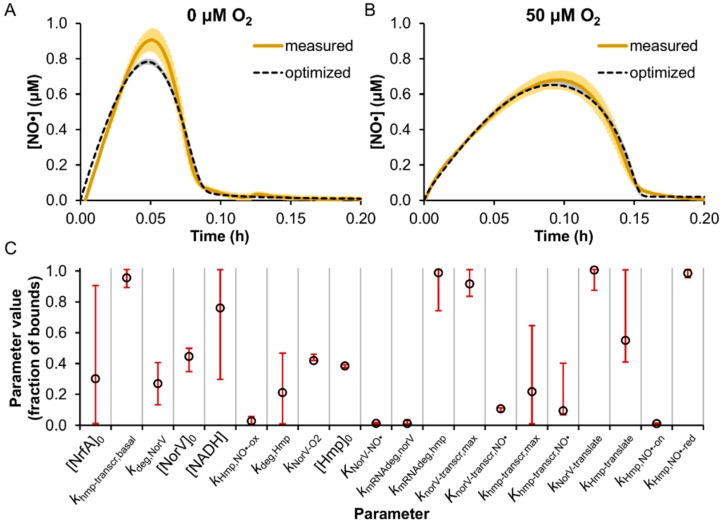
Model training on [NO·] measurements in enterohemorrhagic *Escherichia coli* (EHEC) cultures at 0 and 50 μM O_2_. TUV *hmp*^+^/*norV*^+^ were treated with 50 μM DPTA NONOate at an OD_600_ of 0.05 under conditions of (**A**) 0 μM O_2_ or (**B**) 50 μM O_2_, and the resulting [NO·] was measured (solid yellow line; mean of 3 independent experiments, with light yellow shading representing the SEM). Simulated [NO·] curves for each condition are shown (dashed black lines), and were obtained using the best-fit parameter set from the model training process, where gray shading represents the range of simulated [NO·] curves from the ensemble of viable parameter sets (ER < 10). (**C**) Parameter values (expressed as a fraction of the allowed bounds) obtained from the MCMC analysis are shown, where open circles are the best-fit parameter set (ER = 1), and error bars represent the viable range (min and max) that maintained an ER < 10. Descriptions of parameter names can be found in Table S4.

Uncertain model parameters (68), defined as those not present in the literature or not sufficiently similar to MG1655, were trained on [NO·] measurements in TUV *hmp*^+^/*norV*^+^ cultures treated with 50 μM DPTA NONOate under microaerobic (50 μM O_2_) and anaerobic (0 μM O_2_) conditions (Table S4). The NO· concentration was monitored continuously (>1 read/sec) following DPTA treatment (Figure 2).

A nonlinear least-squares minimization was performed to determine parameter values yielding the minimum sum of the squared residuals (SSR) between the simulated and measured [NO·] at 0 and 50 μM O_2_ (see Section 2). To perform an efficient MCMC search of the parameter space [51], a follow-up sensitivity analysis was executed to assess which parameters had an appreciable impact on [NO·] dynamics, whereby each of the parameters were individually varied among 100 evenly spaced values spanning their allowed range. Parameters whose variation resulted in more than a 5% increase in the SSR (Figure S3) were explored further using the MCMC approach (Figure 2C and Table S4) (see Section 2).

The parameters found to exert the greatest impact on the agreement between simulated and measured [NO·] curves (SSR) were primarily associated with Hmp and NorV, which was expected given that these enzymes are known to be the dominant *E. coli* NO· detoxification systems under oxygenated and anaerobic conditions, respectively [21,22,23,24,27,57,58,59]. Concentrations of NrfA and NADH were also found to impact the SSR, though to a lesser extent relative to Hmp- and NorV-related parameters, and the MCMC analysis revealed that their values were only mildly constrained.

### 3.3. Prediction of EHEC NO· Detoxification Dynamics in the Absence of Hmp or NorV

**Figure 3:**
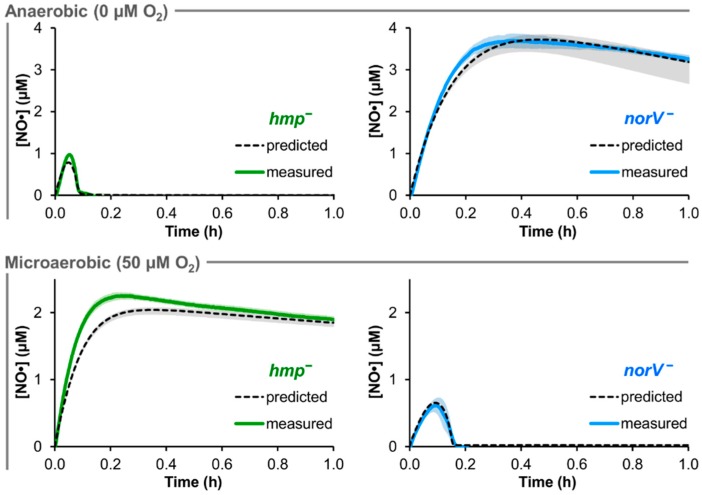
Comparison of predicted and measured [NO·] at 0 and 50 μM O_2_ for *hmp*^−^ and *norV*^−^ EHEC. 50 μM DPTA NONOate treatment was simulated for conditions of 0 and 50 μM O_2_ in cultures of TUV *hmp*^−^/*norV*^+^ and *hmp*^+^/*norV*^−^ at an OD_600_ of 0.05, where the black dashed line was obtained using the best-fit parameter set, and gray shading represents prediction uncertainty (range of viable parameter sets with ER < 10). The corresponding experiments for each condition and mutant were performed, and are shown as solid green (*hmp*^−^/*norV*^+^) or solid blue (*hmp*^+^/*norV*^−^) curves (mean of 3 independent experiments, with light shading of the same color representing the SEM).

The predictive accuracy of the trained EHEC model was assessed with genetic removal of the two major NO· detoxification enzymes, Hmp and NorV. The genes were deleted synthetically (in the model) by setting their concentration and transcription rate to zero. Using the ensemble of parameter sets that sufficiently captured the TUV *hmp*^+^/*norV*^+^ [NO·] curves (ER < 10), NO· treatment (50 μM DPTA NONOate) was simulated for cultures of TUV *hmp*^−^/*norV*^+^ and *hmp*^+^/*norV*^−^ in environments of 0 and 50 μM O_2_ (Figure 3).

Under anaerobic (0 μM O_2_) conditions, NO· consumption by the *norV*^−^ EHEC was predicted to be largely negligible, while the simulated *hmp*^−^ [NO·] was virtually identical to that of the *hmp*^+^/*norV*^+^ strain. Prediction uncertainty was relatively low for *hmp*^−^ at 0 μM O_2_ (<0.05 μM variation in simulated [NO·]), but was slightly heightened for *norV*^−^ (maximum variation of ~0.6 μM NO·). For the 50 μM O_2_ conditions, the predicted behavior for the two mutants was exchanged, where *norV*^−^ simulations now exhibited NO· clearance similar to that of TUV *hmp*^+^/*norV*^+^, while NO· detoxification was severely impaired in *hmp*^−^ cultures. Simulations exhibited little uncertainty in [NO·] for either mutant at 50 μM O_2_, with ≤0.1 μM variation in predicted [NO·].

Corresponding experimental NO· treatment assays were performed with TUV *hmp*^−^/*norV*^+^ and *hmp*^+^/*norV*^−^ cultures at 0 and 50 μM O_2_. The resulting measured [NO·] curves were in excellent agreement with model predictions for both mutants under both O_2_ conditions (Figure 3). Experimental confirmation of the quantitative accuracy in predicted NO· dynamics for mutants lacking either of the two major NO· detoxification systems in EHEC demonstrated a successful translation of the NO· kinetic modeling approach to this medically-relevant pathogen.

### 3.4. Predicted Distribution of NO· Consumption

**Figure 4:**
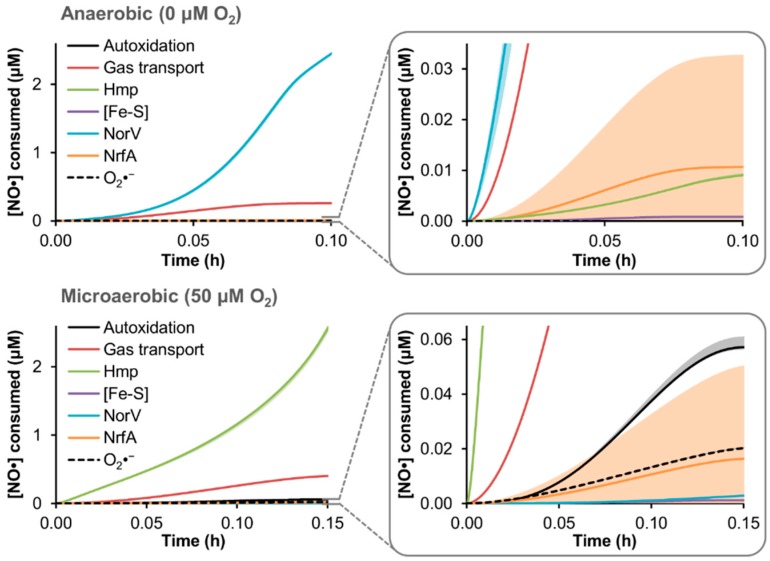
Simulated distribution of NO· consumption in an EHEC culture. The model was used to simulate treatment of a TUV *hmp*^+^/*norV*^+^ culture at an OD_600_ of 0.05 with 50 μM DPTA NONOate under conditions of 0 or 50 μM O_2_, and the resulting distribution of cumulative NO· consumption among the available pathways is shown, up to the approximate time when NO· was cleared (<0.1 μM NO·) from the culture. Lines are predictions obtained using the best-fit parameter set, while lighter shading of a similar color represent prediction uncertainty (range of viable parameter sets with ER < 10). “Autoxidation” is NO· autoxidation, “Gas transport” is loss of NO· to the gas phase, “Hmp” is Hmp-mediated NO· detoxification, “[Fe-S]” is nitrosylation of iron-sulfur clusters, “NorV” is NorV-mediated NO· detoxification, “NrfA” is NrfA-mediated detoxification, and “O_2_·^−^” is reaction of NO· with superoxide.

Upon confirming that model simulations could quantitatively capture EHEC NO· detoxification dynamics under two different O_2_ regimes, and accurately predict the behavior of genetic mutants lacking either of the major NO· defense systems (TUV *hmp*^−^/*norV*^+^ and *hmp*^+^/*norV*^−^), we sought to use the model to quantify the distribution of NO· flux through the reaction network in TUV *hmp*^+^/*norV*^+^ cultures treated with 50 μM DPTA NONOate. DPTA treatment was simulated for conditions of 0 and 50 μM O_2_, and the resulting cumulative NO· consumption by each of the available pathways was quantified (Figure 4).

Consistent with NO· detoxification measured in TUV *hmp*^−^ and *norV*^−^ cultures, the dominant NO· consumption pathway at 0 and 50 μM O_2_ was predicted to be NorV and Hmp, respectively, accounting for over 80% of NO· detoxification up to the time NO· was cleared (<0.1 μM NO·). The majority of the remaining NO· flux for both conditions was loss to the gas phase (~10%–15% of NO· consumption), whereas all other pathways (autoxidation, iron-sulfur cluster ([Fe-S]) nitrosylation, NrfA detoxification, and reaction with superoxide (O_2_·^−^)) did not exceed 2% of the cumulative NO· consumption. Although NO· autoxidation and reaction with O_2_·^−^ was absent under anaerobic conditions due to the lack of O_2_, their combined contribution was predicted to remain modest (<2.5% of the NO· flux) even at 50 μM O_2_. Nitrosylation of [Fe-S] had a largely negligible impact on NO· under either O_2_ condition, accounting for less than 0.1% of the NO· consumed.

From the simulation results, it was clear that the greatest level of relative uncertainty was regarding the participation of NrfA. Since one of the NrfA parameters (initial concentration) was identified as having an impact on the [NO·] curve during the model training procedure (Figure S3), and the MCMC analysis revealed a mild constraint of its value (Figure 2C), it was not surprising that its predicted contribution to NO· consumption would be highly variable among the ensemble of viable models. Further model training on *nrfA*^−^ mutant data could resolve the uncertainty; however, given its minimal contribution to NO· detoxification under either 0 or 50 μM O_2_ conditions (Figure 3), increased resolution of the contribution of NrfA under the conditions used here was not necessary.

## 4. Discussion 

EHEC is a potentially life-threatening pathogen with an extremely low infectious dose (<50 bacteria [13,60,61]). Antibiotic treatments are not recommended due to their exacerbation of Stx-related damage, which can increase the risk for HUS development [3,11,17,18,62]. Although alternative treatment approaches are being explored, such as the development of vaccines or Stx-targeting strategies, they are hindered by a lack of animal models capable of accurately mimicking a human EHEC infection, and the possibility of toxins other than Stx contributing to HUS, respectively [63]. Furthermore, efforts to neutralize the toxins after they have been produced may not be quick enough to prevent damage, whereas inhibition of toxin production would halt damage at the source [11]. Given that EHEC harboring an active NorV exhibit increased Stx production within macrophages [20], therapeutics designed to target this NO· defense system are an attractive solution. Recent studies have shown that NO· stress can decrease Stx production [35], and EHEC lacking NO· detoxification machinery exhibit attenuated survival within macrophages [20]. Here, we have developed and experimentally validated a computational tool to enable quantitative investigation of the broad and complex NO· response network in EHEC.

We previously constructed a kinetic model of the bacterial NO· response of *E. coli* K-12 MG1655, which accounted for the complex biochemical reaction network associated with NO· stress, including iron-sulfur cluster damage, thiol nitrosation, DNA deamination, enzymatic detoxification, and reversible respiratory inhibition [27]. Model predictions were validated with experimental measurements of biochemical species concentrations (NO·, O_2_, NO_2_^−^, and NO_3_^−^), and novel network dynamics were revealed, such as the reduced utility of Hmp with increasing NO· delivery rate [27]. In addition, the model has been used to aid in characterizing the relationship between NO· payload, release rate, and cytotoxicity [42], as well as elucidating the mechanism underlying the enhanced NO· sensitivity of an *E. coli* mutant lacking the ClpP protease [43]. Furthermore, this approach was demonstrated to be an effective tool for studying the dynamics of a different broadly reactive metabolite, hydrogen peroxide (H_2_O_2_) [48].

Here, the NO· kinetic model was adapted to an *E. coli* O157:H7 strain, and trained on experimental data. The adapted model exhibited excellent agreement with [NO·] dynamics measured in EHEC cultures following treatment with the NO·-releasing compound, DPTA NONOate, under both microaerobic and anaerobic conditions (50 and 0 μM O_2_, respectively). Forward predictions of [NO·] in mutant EHEC cultures lacking the major aerobic (Hmp) or anaerobic (NorV) NO· detoxification enzyme at both 0 and 50 μM O_2_ were in excellent agreement with the corresponding experimental measurements, which demonstrated the accuracy and versatility of the translated model.

These results, which demonstrated that Hmp and NorV dominate NO· detoxification in EHEC in the presence and absence of O_2_, respectively, are consistent with previous studies of *E. coli* NO· detoxification [20,21,22,25,57,64]. The NO· dioxygenase function of Hmp requires O_2_ as a substrate, and is therefore inactive under anaerobic conditions [23,57]. Hmp has been demonstrated to harbor an additional, O_2_-indepedent NO· reductase activity, but the rate of this reaction is orders of magnitude slower than the dioxygenation reaction or NorV-mediated NO· reduction [64]. NorV, which is capable of reducing NO· under anaerobic conditions, possesses an O_2_-sensitive flavodiiron catalytic site that is rapidly inactivated upon exposure to oxygenated environments [23]. Although both *hmp* and *norV* genes are expressed in response to NO· exposure in the presence or absence of O_2_ [43,65,66], the substrate requirements and O_2_ sensitivity of their catalytic activity limits the function of these enzymes in each other’s respective O_2_ environments. However, the intermediate regime between 0 and 50 μM O_2_ is far less studied, and the quantitative contribution of Hmp and NorV under these conditions remains ill-defined.

We envision that the computational tool developed here will facilitate future quantitative investigations of the complex NO· stress network in EHEC. For example, mutations found to enhance the NO· sensitivity of EHEC could be interrogated for their underlying mechanism using a model-guided approach, as demonstrated for Δ*clpP* in *E. coli* K-12 [43]. Given the breadth and complexity of the NO· biochemical network, an NO·-sensitive phenotype could arise through an immense number of possible mechanisms. By using the model as a framework to interpret the perturbed dynamics of a pathogen’s NO· response, it could elucidate the specific network components and/or functions involved in the altered behavior, or at least reduce the amount of feasible mechanisms to an experimentally-tractable number. In addition, such a model-guided approach could be translated to the mechanistic investigation of chemical perturbations to the NO· stress network. High-throughput chemical screens could be performed to identify compounds that selectively impair EHEC NO· defenses, and the model could provide a quantitative framework to guide investigations of the compounds’ mechanisms of action. Further, the model could be expanded to incorporate processes governing Stx production, and reveal important relationships between NO· stress and EHEC’s primary virulence factors. Indeed, it has already been shown that NO· interferes with Stx production in EHEC [35], and therefore, a deeper understanding of how these two systems interact could reveal novel treatment strategies. 

## 5. Conclusions

Since antibiotics are not recommended for treatment of EHEC infections due to their enhancement of Stx-related damage, alternative treatment approaches, such as those that target virulence factors, are needed. NO· detoxification is a newly identified virulence system for EHEC [7,20], and the computational tool developed in this work will enable quantitative understanding of NO· stress in EHEC to be gained. Such knowledge could lead to novel anti-infective modalities for the treatment of EHEC, which are sorely needed for this dangerous pathogen. Indeed, continued outbreaks of EHEC offer frequent reminders that our current therapeutic options are insufficient, and novel approaches, such as targeting its NO· defenses, are required to combat this potentially deadly pathogen.

## Supplementary Materials

The following are available online at http://www.mdpi.com/2306-5354/3/1/9/s1, Figure S1: Training of extracellular model parameters. 50 μM DPTA NONOate was delivered to cell-free media (MOPS minimal media with 10 mM glucose) at 50 and 0 μM O_2_, and [NO·] was measured (solid red lines; mean of 3 independent experiments, with light red shading representing the SEM). For the 0 μM O_2_ condition, the media pH was adjusted to 7.2 (from 7.4) with HCl, to mimic the slightly acidified conditions measured in the bioreactor culture for anaerobic assays with EHEC. Model parameters specific to the experimental apparatus and media conditions (*k*_NO·-O2_, *k*_L_*a*_NO·_, and *k*_NONOate_) were optimized on the [NO·] curve measured at 50 μM O_2_ (dashed black line using the best-fit parameter set, with gray shading representing the range of viable parameter sets with ER < 10). Since a decrease in pH increases the rate of NONOate dissociation, the *k*_NONOate_ parameter was released and trained on [NO·] measured in the pH-adjusted media at 0 μM O_2_. The simulation result using the trained *k*_NONOate_ parameter is shown (dashed black line, obtained using the best-fit parameter value, with gray shading representing viable parameter values with ER < 10), Figure S2: Training of parameters associated with the respiratory module. TUV *hmp*^+^/*norV*^+^ in mid-exponential phase were delivered to the bioreactor to an OD_600_ of 0.05, and the O_2_ consumption was measured (solid purple line; mean of 3 independent experiments with light purple shading representing the SEM). Model parameters associated with the aerobic respiratory module (NADH dehydrogenases and cytochrome ubiquinol oxidases) were trained on the measured [O_2_]. The simulated [O_2_] generated by the trained model is shown (dashed black line was obtained using the best-fit parameter set, with gray shading representing the range of viable parameter sets with ER < 10), Figure S3: Impact of individual parameter variation on [NO·] distribution. A sensitivity analysis was performed on the 68 parameters trained on the [NO·] curves measured in TUV *hmp*^+^/*norV*^+^ cultures following treatment with 50 μM DPTA NONOate at 0 and 50 μM O_2_. Each parameter was individually varied within its permitted bounds, and the resulting increase in SSR was quantified (fold increase = SSR/SSR_0_). Shown is the maximum fold increase in SSR achieved for each parameter, showing only the 20 parameters exhibiting a maximum increase of ≥5% (1.05-fold), Table S1: BLAST analysis. Proteins with <99% amino acid (AA) similarity are in bold, Table S2: Training of extracellular model parameters. Parameter values were released and allowed to vary within the “Minimum” and “Maximum” bounds. Optimal (best-fit, yielding minimum SSR between measured and simulated [NO·]) parameter values are reported, along with their confidence interval (CI), defined as the range of the parameter among viable parameter sets with ER < 10, Table S3: Training of model parameters associated with the respiratory module. Parameter values were released and allowed to vary within the “Minimum” and “Maximum” bounds. Optimal (best-fit, yielding minimum SSR between measured and simulated [O_2_]) parameter values are reported, along with their confidence interval (CI), defined as the range of the parameter among viable parameter sets with ER < 10, Table S4: Training of organism-specific model parameters. Parameter values were released and allowed to vary within the “Minimum” and “Maximum” bounds. Optimal (best-fit, yielding minimum SSR between measured and simulated [NO·]) parameter values are reported, along with their confidence interval (CI), defined as the range of the parameter among viable parameter sets with ER < 10. Only the 20 parameters identified as having a substantial impact on the SSR (>5% increase) are shown.

## References

[1] Russo T.A., Johnson J.R. (2000). Proposal for a new inclusive designation for extraintestinal pathogenic isolates of *Escherichia coli*: ExPEC. J. Infect. Dis..

[2] Wiles T.J., Kulesus R.R., Mulvey M.A. (2008). Origins and virulence mechanisms of uropathogenic *Escherichia coli*. Exp. Mol. Pathol..

[3] Nataro J.P., Kaper J.B. (1998). Diarrheagenic *Escherichia coli*. Clin. Microbiol. Rev..

[4] Kaper J.B., Nataro J.P., Mobley H.L. (2004). Pathogenic *Escherichia coli*. Nat. Rev. Microbiol..

[5] Flores-Mireles A.L., Walker J.N., Caparon M., Hultgren S.J. (2015). Urinary tract infections: Epidemiology, mechanisms of infection and treatment options. Nat. Rev. Microbiol..

[6] Michino H., Araki K., Minami S., Takaya S., Sakai N., Miyazaki M., Ono A., Yanagawa H. (1999). Massive outbreak of *Escherichia coli* O157:H7 infection in schoolchildren in Sakai City, Japan, associated with consumption of white radish sprouts. Am. J. Epidemiol..

[7] Kulasekara B.R., Jacobs M., Zhou Y., Wu Z.N., Sims E., Saenphimmachak C., Rohmer L., Ritchie J.M., Radey M., McKevitt M. (2009). Analysis of the genome of the *Escherichia coli* O157:H7 2006 spinach-associated outbreak isolate indicates candidate genes that may enhance virulence. Infect. Immun..

[8] Centers for Disease Control and Prevention (2006). Ongoing multistate outbreak of *Escherichia coli* serotype O157:H7 infections associated with consumption of fresh spinach—United States, September 2006. MMWR Morb. Mortal. Wkly. Rep..

[9] Frank C., Werber D., Cramer J.P., Askar M., Faber M., an der Heiden M., Bernard H., Fruth A., Prager R., Spode A. (2011). Epidemic profile of Shiga-toxin-producing *Escherichia coli* O104:H4 outbreak in Germany. N. Engl. J. Med..

[10] Mellmann A., Harmsen D., Cummings C.A., Zentz E.B., Leopold S.R., Rico A., Prior K., Szczepanowski R., Ji Y.M., Zhang W.L. (2011). Prospective genomic characterization of the German enterohemorrhagic *Escherichia coli* O104:H4 outbreak by rapid next generation sequencing technology. PLoS ONE.

[11] Goldwater P.N., Bettelheim K.A. (2012). Treatment of enterohemorrhagic *Escherichia coli* (EHEC) infection and hemolytic uremic syndrome (HUS). BMC Med..

[12] Torres A.G., Zhou X., Kaper J.B. (2005). Adherence of diarrheagenic *Escherichia coli* strains to epithelial cells. Infect. Immun..

[13] Kaper J.B., Karmali M.A. (2008). The continuing evolution of a bacterial pathogen. Proc. Natl. Acad. Sci. USA.

[14] Tarr P.I., Gordon C.A., Chandler W.L. (2005). Shiga-toxin-producing *Escherichia coli* and haemolytic uraemic syndrome. Lancet.

[15] Obrig T.G., Moran T.P., Brown J.E. (1987). The mode of action of Shiga toxin on peptide elongation of eukaryotic protein synthesis. Biochem. J..

[16] Schuller S., Heuschkel R., Torrente F., Kaper J.B., Phillips A.D. (2007). Shiga toxin binding in normal and inflamed human intestinal mucosa. Microbes Infect..

[17] Zhang X.P., McDaniel A.D., Wolf L.E., Keusch G.T., Waldor M.K., Acheson D.W.K. (2000). Quinolone antibiotics induce shiga toxin-encoding bacteriophages, toxin production, and death in mice. J. Infect. Dis..

[18] Bielaszewska M., Idelevich E.A., Zhang W., Bauwens A., Schaumburg F., Mellmann A., Peters G., Karch H. (2012). Effects of antibiotics on Shiga toxin 2 production and bacteriophage induction by epidemic *Escherichia coli* O104:H4 strain. Antimicrob. Agents Chemother..

[19] Garcia-Angulo V.A., Kalita A., Torres A.G. (2013). Advances in the development of enterohemorrhagic *Escherichia coli* vaccines using murine models of infection. Vaccine.

[20] Shimizu T., Tsutsuki H., Matsumoto A., Nakaya H., Noda M. (2012). The nitric oxide reductase of enterohaemorrhagic *Escherichia coli* plays an important role for the survival within macrophages. Mol. Microbiol..

[21] Gomes C.M., Giuffre A., Forte E., Vicente J.B., Saraiva L.M., Brunori M., Teixeira M. (2002). A novel type of nitric-oxide reductase. *Escherichia coli* flavorubredoxin. J. Biol. Chem..

[22] Gardner A.M., Helmick R.A., Gardner P.R. (2002). Flavorubredoxin, an inducible catalyst for nitric oxide reduction and detoxification in *Escherichia coli*. J. Biol. Chem..

[23] Gardner A.M., Gardner P.R. (2002). Flavohemoglobin detoxifies nitric oxide in aerobic, but not anaerobic, *Escherichia coli*—Evidence for a novel inducible anaerobic nitric oxide-scavenging activity. J. Biol. Chem..

[24] Vine C.E., Cole J.A. (2011). Nitrosative stress in *Escherichia coli*: Reduction of nitric oxide. Biochem. Soc. Trans..

[25] Gardner P.R., Gardner A.M., Martin L.A., Salzman A.L. (1998). Nitric oxide dioxygenase: An enzymic function for flavohemoglobin. Proc. Natl. Acad. Sci. USA.

[26] Hausladen A., Gow A.J., Stamler J.S. (1998). Nitrosative stress: Metabolic pathway involving the flavohemoglobin. Proc. Natl. Acad. Sci. USA.

[27] Robinson J.L., Brynildsen M.P. (2013). A kinetic platform to determine the fate of nitric oxide in *Escherichia coli*. PLoS Comput. Biol..

[28] Robinson J.L., Adolfsen K.J., Brynildsen M.P. (2014). Deciphering nitric oxide stress in bacteria with quantitative modeling. Curr. Opin. Microbiol..

[29] Svensson L., Poljakovic M., Save S., Gilberthorpe N., Schon T., Strid S., Corker H., Poole R.K., Persson K. (2010). Role of flavohemoglobin in combating nitrosative stress in uropathogenic *Escherichia coli*—Implications for urinary tract infection. Microb. Pathog..

[30] Bang I.S., Liu L., Vazquez-Torres A., Crouch M.L., Stamler J.S., Fang F.C. (2006). Maintenance of nitric oxide and redox homeostasis by the *Salmonella* flavohemoglobin hmp. J. Biol. Chem..

[31] Stevanin T.M., Poole R.K., Demoncheaux E.A.G., Read R.C. (2002). Flavohemoglobin Hmp protects *Salmonella enterica* serovar typhimurium from nitric oxide-related killing by human macrophages. Infect. Immun..

[32] Sebbane F., Lemaitre N., Sturdevant D.E., Rebeil R., Virtaneva K., Porcella S.F., Hinnebusch B.J. (2006). Adaptive response of *Yersinia pestis* to extracellular effectors of innate immunity during bubonic plague. Proc. Natl. Acad. Sci. USA.

[33] Richardson A.R., Dunman P.M., Fang F.C. (2006). The nitrosative stress response of *Staphylococcus aureus* is required for resistance to innate immunity. Mol. Microbiol..

[34] Stern A.M., Hay A.J., Liu Z., Desland F.A., Zhang J., Zhong Z.T., Zhu J. (2012). The NorR regulon is critical for *Vibrio cholerae* resistance to nitric oxide and sustained colonization of the intestines. mBio.

[35] Vareille M., de Sablet T., Hindre T., Martin C., Gobert A.P. (2007). Nitric oxide inhibits Shiga-toxin synthesis by enterohemorrhagic *Escherichia coli*. Proc. Natl. Acad. Sci. USA.

[36] Branchu P., Matrat S., Vareille M., Garrivier A., Durand A., Crepin S., Harel J., Jubelin G., Gobert A.P. (2014). NsrR, GadE, and GadX interplay in repressing expression of the *Escherichia coli* O157: H7 LEE pathogenicity island in response to nitric oxide. PLoS Pathog..

[37] Schomburg I., Chang A., Placzek S., Sohngen C., Rother M., Lang M., Munaretto C., Ulas S., Stelzer M., Grote A. (2013). BRENDA in 2013: Integrated reactions, kinetic data, enzyme function data, improved disease classification: New options and contents in BRENDA. Nucleic Acids Res..

[38] Gardner P.R., Gardner A.M., Martin L.A., Dou Y., Li T., Olson J.S., Zhu H., Riggs A.F. (2000). Nitric-oxide dioxygenase activity and function of flavohemoglobins. sensitivity to nitric oxide and carbon monoxide inhibition. J. Biol. Chem..

[39] Bowman L.A., McLean S., Poole R.K., Fukuto J.M. (2011). The diversity of microbial responses to nitric oxide and agents of nitrosative stress close cousins but not identical twins. Adv. Microb. Physiol..

[40] Lancaster J.R. (2006). Nitroxidative, nitrosative, and nitrative stress: Kinetic predictions of reactive nitrogen species chemistry under biological conditions. Chem. Res. Toxicol..

[41] Lim C.H., Dedon P.C., Deen W.M. (2008). Kinetic analysis of intracellular concentrations of reactive nitrogen species. Chem. Res. Toxicol..

[42] Robinson J.L., Brynildsen M.P. (2014). Model-driven identification of dosing regimens that maximize the antimicrobial activity of nitric oxide. Metab. Eng. Commun..

[43] Robinson J.L., Brynildsen M.P. (2015). An ensemble-guided approach Identifies ClpP as a major regulator of transcript levels in nitric oxide-stressed *Escherichia coli*. Metab. Eng..

[44] Campellone K.G., Giese A., Tipper D.J., Leong J.M. (2002). A tyrosine-phosphorylated 12-amino-acid sequence of enteropathogenic *Escherichia coli* Tir binds the host adaptor protein Nck and is required for Nck localization to actin pedestals. Mol. Microbiol..

[45] Zaslaver A., Bren A., Ronen M., Itzkovitz S., Kikoin I., Shavit S., Liebermeister W., Surette M.G., Alon U. (2006). A comprehensive library of fluorescent transcriptional reporters for *Escherichia coli*. Nat. Methods.

[46] Datsenko K.A., Wanner B.L. (2000). One-step inactivation of chromosomal genes in *Escherichia coli* K-12 using PCR products. Proc. Natl. Acad. Sci. USA.

[47] Baba T., Ara T., Hasegawa M., Takai Y., Okumura Y., Baba M., Datsenko K.A., Tomita M., Wanner B.L., Mori H. (2006). Construction of *Escherichia coli* K-12 in-frame, single-gene knockout mutants: The Keio collection. Mol. Syst. Biol..

[48] Adolfsen K.J., Brynildsen M.P. (2015). A kinetic platform to determine the fate of hydrogen peroxide in *Escherichia coli*. PLoS Comput. Biol..

[49] Denicola A., Souza J.M., Radi R., Lissi E. (1996). Nitric oxide diffusion in membranes determined by fluorescence quenching. Arch. Biochem. Biophys..

[50] Kelm M. (1999). Nitric oxide metabolism and breakdown. Biochim. Biophys. Acta.

[51] Zamora-Sillero E., Hafner M., Ibig A., Stelling J., Wagner A. (2011). Efficient characterization of high-dimensional parameter spaces for systems biology. BMC Syst. Biol..

[52] Akaike H. (1973). Information theory and extension of the maximum likelihood principle. Proceedings of the 2nd International Symposium on Information Theory.

[53] Hurvich C.M., Tsai C.L. (1989). Regression and time-series model selection in small samples. Biometrika.

[54] Turkheimer F.E., Hinz R., Cunningham V.J. (2003). On the undecidability among kinetic models: From model selection to model averaging. J. Cereb. Blood Flow Metab..

[55] Burnham K.P., Anderson D.R. (2002). Model Selection and Multimodel Inference: A Practical Information-Theoretic Approach.

[56] Caspi R., Billington R., Ferrer L., Foerster H., Fulcher C.A., Keseler I.M., Kothari A., Krummenacker M., Latendresse M., Mueller L.A. (2015). The MetaCyc database of metabolic pathways and enzymes and the BioCyc collection of pathway/genome databases. Nucleic Acids Res..

[57] Hausladen A., Gow A., Stamler J.S. (2001). Flavohemoglobin denitrosylase catalyzes the reaction of a nitroxyl equivalent with molecular oxygen. Proc. Natl. Acad. Sci. USA.

[58] Mills C.E., Sedelnikova S., Soballe B., Hughes M.N., Poole R.K. (2001). *Escherichia coli* flavohaemoglobin (Hmp) with equistoichiometric FAD and haem contents has a low affinity for dioxygen in the absence or presence of nitric oxide. Biochem. J..

[59] Stevanin T.M., Ioannidis N., Mills C.E., Kim S.O., Hughes M.N., Poole R.K. (2000). Flavohemoglobin Hmp affords inducible protection for *Escherichia coli* respiration, catalyzed by cytochromes *bo*’ or *bd*, from nitric oxide. J. Biol. Chem..

[60] Tilden J., Young W., McNamara A.M., Custer C., Boesel B., LambertFair M., Majkowski J., Vugia D., Werner S.B., Hollingsworth J. (1996). A new route of transmission for *Escherichia coli*: Infection from dry fermented salami. Am. J. Public Health.

[61] Tuttle J., Gomez T., Doyle M.P., Wells J.G., Zhao T., Tauxe R.V., Griffin P.M. (1999). Lessons from a large outbreak of *Escherichia coli* O157:H7 infections: Insights into the infectious dose and method of widespread contamination of hamburger patties. Epidemiol. Infect..

[62] Wong C.S., Jelacic S., Habeeb R.L., Watkins S.L., Tarr P.I. (2000). The risk of the hemolytic-uremic syndrome after antibiotic treatment of *Escherichia coli* O157:H7 infections. N. Engl. J. Med..

[63] Orth D., Grif K., Zimmerhackl L.B., Wurzner R. (2008). Prevention and treatment of enterohemorrhagic *Escherichia coli* infections in humans. Expert Rev. Anti Infect Ther..

[64] Gardner A.M., Martin L.A., Gardner P.R., Dou Y., Olson J.S. (2000). Steady-state and transient kinetics of *Escherichia coli* nitric-oxide dioxygenase (flavohemoglobin). The B10 tyrosine hydroxyl is essential for dioxygen binding and catalysis. J. Biol. Chem..

[65] Hyduke D.R., Jarboe L.R., Tran L.M., Chou K.J., Liao J.C. (2007). Integrated network analysis identifies nitric oxide response networks and dihydroxyacid dehydratase as a crucial target in *Escherichia coli*. Proc. Natl. Acad. Sci. USA.

[66] Pullan S.T., Gidley M.D., Jones R.A., Barrett J., Stevanin T.A., Read R.C., Green J., Poole R.K. (2007). Nitric oxide in chemostat-cultured *Escherichia coli* is sensed by Fnr and other global regulators: Unaltered methionine biosynthesis indicates lack of S nitrosation. J. Bacteriol..

