# Supplementary material for: Construction and Experimental Validation of a Quantitative Kinetic Model of Nitric Oxide Stress in Enterohemorrhagic Escherichia coli O157:H7
Source: Bioengineering (Basel). 2016 Feb 6;3(1):9. doi: 10.3390/bioengineering3010009 (PMC5597167; doi:10.3390/bioengineering3010009)
Supplement: Supplementary file 1 [file bioengineering-03-00009-s001.pdf]

Supplementary Materials: Construction and Experimental Validation of a Quantitative Kinetic Model of Nitric Oxide Stress in Enterohemorrhagic *Escherichia coli* O157:H7

Jonathan L. Robinson and Mark P. Brynildsen

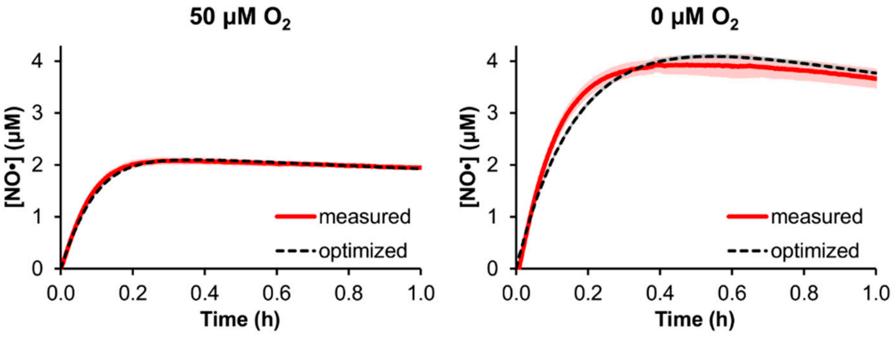

Figure S1. Training of extracellular model parameters. 50 µM DPTA NONOate was delivered to cell-free media (MOPS minimal media with 10 mM glucose) at 50 and 0 µM O_2, and [NO·] was measured (solid red lines; mean of 3 independent experiments, with light red shading representing the SEM). For the 0 µM O_2 condition, the media pH was adjusted to 7.2 (from 7.4) with HCl, to mimic the slightly acidified conditions measured in the bioreactor culture for anaerobic assays with EHEC. Model parameters specific to the experimental apparatus and media conditions ($k_{NO\cdot\text{-}O2}$, $k_{La_{NO\cdot}}$, and $k_{NONOate}$) were optimized on the [NO·] curve measured at 50 µM O_2 (dashed black line using the best-fit parameter set, with gray shading representing the range of viable parameter sets with ER < 10). Since a decrease in pH increases the rate of NONOate dissociation, the $k_{NONOate}$ parameter was released and trained on [NO·] measured in the pH-adjusted media at 0 µM O_2. The simulation result using the trained $k_{NONOate}$ parameter is shown (dashed black line, obtained using the best-fit parameter value, with gray shading representing viable parameter values with ER < 10).

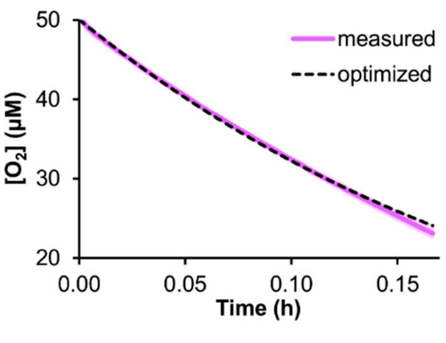

Figure S2. Training of parameters associated with the respiratory module. TUV *hmp*⁺/*norV*⁺ in mid-exponential phase were delivered to the bioreactor to an OD_{600} of 0.05, and the O_2 consumption was measured (solid purple line; mean of 3 independent experiments with light purple shading representing the SEM). Model parameters associated with the aerobic respiratory module (NADH dehydrogenases and cytochrome ubiquinol oxidases) were trained on the measured [O_2]. The simulated [O_2] generated by the trained model is shown (dashed black line was obtained using the best-fit parameter set, with gray shading representing the range of viable parameter sets with ER < 10).

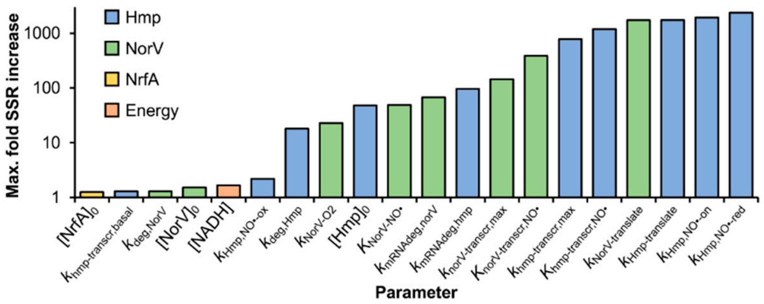

Figure S3. Impact of individual parameter variation on [NO·] distribution. A sensitivity analysis was performed on the 68 parameters trained on the [NO·] curves measured in TUV *hmp*$^+$/*norV*$^+$ cultures following treatment with 50 μM DPTA NONOate at 0 and 50 μM O_2. Each parameter was individually varied within its permitted bounds, and the resulting increase in SSR was quantified (fold increase = SSR/SSR_0). Shown is the maximum fold increase in SSR achieved for each parameter, showing only the 20 parameters exhibiting a maximum increase of ≥5% (1.05-fold).

Table S1. BLAST analysis. Proteins with <99% amino acid (AA) similarity are in bold.

Protein		AA Similarity		Description
MG1655	EDL933	EDL/MG	%	
IscU	[Z3796]	128/128	100.0%	scaffold protein for iron-sulfur cluster assembly
IscS	YfhO	404/404	100.0%	cysteine desulfurase
LigA	Lig	664/671	99.0%	DNA ligase
PolA	PolA	927/928	99.9%	DNA polymerase I
**XthA**	**XthA**	**263/268**	**98.1%**	**exonuclease III**
GS-FDH (FrmA)	AdhC	367/369	99.5%	glutathione-dependent formaldehyde dehydrogenase
GOR	Gor	446/450	99.1%	gluthathione reductase
TrxA	TrxA	109/109	100.0%	thioredoxin 1
TrxC	TrxC	139/139	100.0%	thioredoxin 2
TrxR	TrxB	321/321	100.0%	thioredoxin reductase
**AlkA**	**AlkA**	**274/282**	**97.2%**	**3-methyl-adenine-DNA glycosylase II**
Ung	Ung	227/229	99.1%	uracil-DNA glycosylase
CyoA	CyoA	312/315	99.0%	cytochrome bo terminal oxidase subunit II
CyoB	CyoB	663/663	100.0%	cytochrome bo terminal oxidase subunit I
CyoC	CyoC	203/204	99.5%	cytochrome bo terminal oxidase subunit III
CyoD	CyoD	109/109	100.0%	cytochrome bo terminal oxidase subunit IV
CydA	CydA	522/522	100.0%	cytochrome bd-I terminal oxidase subunit I
CydB	CydB	379/379	100.0%	cytochrome bd-I terminal oxidase subunit II
Hmp	HmpA	395/396	99.7%	nitric oxide dioxygenase
**NorV**	**[Z4018]**	**409/479**	**85.4%**	**flavorubredoxin (nitric oxide reductase)**
**NorW**	**YgbD**	**369/377**	**97.9%**	**flavorubredoxin reductase**
SodA	SodA	205/206	99.5%	superoxide dismutase (Mn)
SodB	SodB	193/193	100.0%	superoxide dismutase (Fe)
SodC	SodC	173/173	100.0%	superoxide dismutase (Cu-Zn)
NrfA	NrfA	477/478	99.8%	formate dependent nitrite reductase (subunit A)
NrfB	NrfB	188/188	100.0%	formate dependent nitrite reductase (penta-heme cytochrome c)
NrfC	NrfC	223/223	100.0%	formate dependent nitrite reductase ([4Fe-4S] subunit)
NrfD	NrfD	315/318	99.1%	formate dependent nitrite reductase (subunit D)
NuoA	NuoA	146/147	99.3%	NADH:ubiquinone oxidoreductase, membrane subunit A
NuoB	NuoB	219/220	99.5%	NADH:ubiquinone oxidoreductase, chain B
NuoC	NuoC	593/596	99.5%	NADH:ubiquinone oxidoreductase, chain CD
NuoE	NuoE	165/166	99.4%	NADH:ubiquinone oxidoreductase, chain E
NuoF	NuoF	444/445	99.8%	NADH:ubiquinone oxidoreductase, chain F
NuoG	NuoG	906/908	99.8%	NADH:ubiquinone oxidoreductase, chain G
NuoH	NuoH	325/325	100.0%	NADH:ubiquinone oxidoreductase, membrane subunit H
NuoI	NuoI	180/180	100.0%	NADH:ubiquinone oxidoreductase, chain I
NuoJ	NuoJ	184/184	100.0%	NADH:ubiquinone oxidoreductase, membrane subunit J
NuoK	NuoK	100/100	100.0%	NADH:ubiquinone oxidoreductase, membrane subunit K
NuoL	NuoL	612/613	99.8%	NADH:ubiquinone oxidoreductase, membrane subunit L
NuoM	NuoM	509/509	100.0%	NADH:ubiquinone oxidoreductase, membrane subunit M
**NuoN**	**NuoN**	**423/485**	**87.2%**	**NADH:ubiquinone oxidoreductase, membrane subunit N**
NDH-2 (Ndh)	Ndh	431/434	99.3%	NADH:quinone oxidoreductase II

Table S2. Training of extracellular model parameters. Parameter values were released and allowed to vary within the "Minimum" and "Maximum" bounds. Optimal (best-fit, yielding minimum SSR between measured and simulated [NO·]) parameter values are reported, along with their confidence interval (CI), defined as the range of the parameter among viable parameter sets with ER < 10.

#	Parameter	Parameter description	Minimum	Maximum	Optimal	CI	Units	Ref.
1	$k_{NONOate}$	DPTA NONOate dissociation rate (pH 7.4)	4.81×10^{-5}	3.85×10^{-4}	5.98×10^{-5}	$(5.87–6.08) \times 10^{-5}$	s^{-1}	[1]
		DPTA NONOate dissociation rate (pH 7.2) *	4.81×10^{-5}	3.85×10^{-4}	7.78×10^{-5}	$(7.69–7.87) \times 10^{-5}$	s^{-1}	[1]
2	$k_La_{NO•}$	Rate of NO· transfer to gas phase	1.00×10^{-4}	1.00×10^{-2}	1.63×10^{-3}	$(1.59–1.68) \times 10^{-3}$	s^{-1}	[a]
3	$k_{NO•\text{-}O2}$	NO· autoxidation rate	9.00×10^{5}	2.40×10^{6}	2.40×10^{6}	$(2.31–2.40) \times 10^{6}$	$M^{-2}s^{-1}$	[2]

* The dissociation rate was also determined for media with a lower pH, as anaerobically grown cells slightly acidified the media (from pH 7.4 to 7.2). [a] The $k_La_{NO•}$ was allowed to vary within one order of magnitude of the O_2 k_La measured for the same experimental apparatus.

Table S3. Training of model parameters associated with the respiratory module. Parameter values were released and allowed to vary within the "Minimum" and "Maximum" bounds. Optimal (best-fit, yielding minimum SSR between measured and simulated [O_2]) parameter values are reported, along with their confidence interval (CI), defined as the range of the parameter among viable parameter sets with ER < 10.

#	Parameter	Parameter description	Minimum	Maximum	Optimal	CI	Units	Ref.
1	$k_{Cyo,cat}$	Cytochrome *bo* terminal oxidase; k_{cat}	18	150	19.6	18.0–23.8	s^{-1}	[3–5]
2	$k_{Cyd,cat}$	Cytochrome *bd* terminal oxidase; k_{cat}	12	470	353	334–414	s^{-1}	[4–6]
3	$k_{NDH1,cat}$	NADH dehydrogenase I; k_{cat}	50	600	537	495–579	s^{-1}	[7,8]
4	$K_{NDH1,Q}$	NADH dehydrogenase I; $K_{d,Q}$	3.00×10^{-6}	3.00×10^{-4}	3.11×10^{-6}	$(3.00–9.23) \times 10^{-6}$	M	[9] [a]
5	$K_{NDH1,NADH}$	NADH dehydrogenase I; $K_{m,NADH}$	7.20×10^{-7}	7.20×10^{-5}	2.62×10^{-5}	$(2.46–2.68) \times 10^{-5}$	M	[8] [b]
6	$K_{NDH1,Q8}$	NADH dehydrogenase I; $K_{m,Q8}$	3.00×10^{-6}	3.00×10^{-4}	3.17×10^{-6}	$(3.00–4.14) \times 10^{-6}$	M	[9] [a]
7	$k_{NDH2,cat}$	NADH dehydrogenase II; k_{cat}	17.1	474	347	293–373	s^{-1}	[10,11]
8	$[Cyo]_0$	Initial concentration of cytochrome *bo*	1.58×10^{-8}	1.58×10^{-6}	2.00×10^{-8}	$(1.59–13.3) \times 10^{-8}$	M	[12] [c]
9	$[Cyd]_0$	Initial concentration of cytochrome *bd*	1.06×10^{-8}	1.06×10^{-6}	1.00×10^{-6}	$(8.54–10.6) \times 10^{-7}$	M	[12] [c]
10	$[Q_8]_0$	Initial concentration of ubiquinone-8	4.48×10^{-5}	4.48×10^{-3}	4.47×10^{-3}	$(4.02–4.48) \times 10^{-3}$	M	[13] [c]
11	$[Q_8H_2]_0$	Initial concentration of ubiquinol-8	4.48×10^{-5}	4.48×10^{-3}	5.36×10^{-5}	$(4.48–5.80) \times 10^{-5}$	M	[13] [c]
12	$[NDH1]_0$	Initial concentration of NADH dehydrogenase I	2.70×10^{-8}	2.70×10^{-6}	1.60×10^{-6}	$(1.46–1.72) \times 10^{-6}$	M	[14] [c]
13	$[NDH2]_0$	Initial concentration of NADH dehydrogenase II	3.05×10^{-9}	3.05×10^{-7}	2.28×10^{-7}	$(1.72–3.05) \times 10^{-7}$	M	[15] [c]

[a] Parameter allowed to vary within one order of magnitude of the value reported for the *E. coli* $K_{m,Q2}$ value. [b] Parameter allowed to vary within one order of magnitude of the value reported for the *E. coli* $K_{m,NADH}$ value. [c] The concentrations of respiratory module components were allowed to vary within one order of magnitude of their reported value. In cases where concentrations were reported as molecules/cell, they were converted to M, assuming a cell volume of 3.2×10^{-15} L [16]. Concentrations of ubiquinone and ubiquinol were converted from units of μmol/g dry cell weight [13] to M, assuming a cell density of 448 g dry cell weight/L [14].

Table S4. Training of organism-specific model parameters. Parameter values were released and allowed to vary within the "Minimum" and "Maximum" bounds. Optimal (best-fit, yielding minimum SSR between measured and simulated [NO·]) parameter values are reported, along with their confidence interval (CI), defined as the range of the parameter among viable parameter sets with ER < 10. Only the 20 parameters identified as having a substantial impact on the SSR (>5% increase) are shown.

#	Parameter	Parameter description	Minimum	Maximum	Optimal	CI	Units	Ref.
1	$k_{Hmp,NO\bullet\text{-}on}$	Hmp detoxification; NO· binding to Hmp	4.0×10^{6}	2.6×10^{7}	4.00×10^{6}	$(4.00–4.11) \times 10^{6}$	$M^{-1}s^{-1}$	[17]
2	$k_{Hmp,NO\bullet\text{-}ox}$	Hmp detoxification; NO· binding to Hmp-O_2	9.6×10^{8}	2.4×10^{9}	9.84×10^{8}	$(9.60–10.3) \times 10^{8}$	$M^{-1}s^{-1}$	[17]
3	$k_{Hmp,NO\bullet\text{-}red}$	Hmp detoxification; NO· reduction	0.013	0.240	0.234	0.228–0.240	s^{-1}	[18]
4	$k_{hmp\text{-}transcr,basal}$	*hmp* transcription; basal rate	0	2.78×10^{-12}	2.63×10^{-12}	$(2.45–2.78) \times 10^{-12}$	$M{\cdot}s^{-1}$	[19–21] [a]
5	$k_{hmp\text{-}transcr,max}$	*hmp* transcription; maximum rate	1.19×10^{-10}	4.57×10^{-10}	1.90×10^{-10}	$(1.20–3.34) \times 10^{-10}$	$M{\cdot}s^{-1}$	[19–21]
6	$K_{hmp\text{-}transcr,NO\bullet}$	*hmp* transcription; NO· dissociation constant	1.0×10^{-8}	1.0×10^{-5}	8.49×10^{-7}	$(5.90–39.3) \times 10^{-7}$	M	[22,23] *b*
7	$k_{mRNAdeg,hmp}$	*hmp* mRNA degradation	3.35×10^{-4}	1.65×10^{-2}	1.61×10^{-2}	$(1.22–1.65) \times 10^{-2}$	s^{-1}	[24,25]
8	$k_{Hmp\text{-}translate}$	Hmp translation	0.057	1.49	0.833	0.631–1.49	s^{-1}	[14,26–28]
9	$k_{deg,Hmp}$	Hmp degradation	1.0×10^{-5}	1.0×10^{-3}	2.10×10^{-4}	$(1.02–46.3) \times 10^{-5}$	s^{-1}	[29,30]
10	$K_{NorV\text{-}NO\bullet}$	NorV detoxification; $K_{m,NO\cdot}$	1.0×10^{-7}	1.0×10^{-6}	1.02×10^{-7}	$(1.00–1.09) \times 10^{-7}$	M	[31,32]
11	$k_{NorV\text{-}O2}$	O_2-mediated NorV inactivation	1.0	1.0×10^{6}	4.09×10^{5}	$(4.09–4.52) \times 10^{5}$	$M^{-1}s^{-1}$	[33,34]
12	$k_{norV\text{-}transcr,max}$	*norV* transcription; maximum rate	1.19×10^{-10}	4.57×10^{-10}	4.26×10^{-10}	$(3.99–4.57) \times 10^{-10}$	$M{\cdot}s^{-1}$	[19–21]
13	$K_{norV\text{-}transcr,NO\bullet}$	*norV* transcription; NO· dissociation constant	1.0×10^{-8}	1.0×10^{-5}	9.82×10^{-7}	$(7.85–10.4) \times 10^{-7}$	M	[22,23] *b*
14	$k_{mRNAdeg,norV}$	*norV* mRNA degradation	3.35×10^{-4}	1.65×10^{-2}	3.58×10^{-4}	$(3.36–7.40) \times 10^{-4}$	s^{-1}	[24,25]
15	$k_{NorV\text{-}translate}$	NorV translation	0.057	1.49	1.49	1.30–1.49	s^{-1}	[14,26–28]
16	$k_{deg,NorV}$	NorV degradation	1.0×10^{-5}	1.0×10^{-3}	2.67×10^{-4}	$(1.32–4.03) \times 10^{-4}$	s^{-1}	[29,30]
17	$[Hmp]_0$	Initial concentration of Hmp	0	1.0×10^{-6}	3.74×10^{-7}	$(3.63–3.83) \times 10^{-7}$	M	[35] [a]
18	$[NorV]_0$	Initial concentration of NorV	0	1.0×10^{-6}	4.36×10^{-7}	$(3.38–4.90) \times 10^{-7}$	M	[c]
19	$[NrfA]_0$	Initial concentration of NrfA	0	1.0×10^{-6}	2.92×10^{-7}	$(2.12–896) \times 10^{-9}$	M	[c]
20	[NADH]	Concentration of NADH	8.30×10^{-6}	8.30×10^{-4}	6.25×10^{-4}	$(2.44–8.29) \times 10^{-4}$	M	[36] [d]

[a] The initial Hmp concentration and basal transcription rates were allowed to respectively vary below 1 μM and 10 nM/h (approximately 100-fold less than the maximum transcription rate), given that Hmp levels are nearly undetectable in unstressed cells [35]. [b] The dissociation rate constant of NO· governing activation of *hmp* and *norV* transcription was permitted to vary within the physiological range reported for NO· (nM to low μM). [c] Initial concentrations of NorV and NrfA were restricted to ≤ 1 μM, given the dependence of their expression on the presence of NO·, NO_2^-, or NO_3^- [37], none of which were present in the growth media prior to DPTA NONOate treatment. [d] The concentration of NADH was permitted to vary within one order of magnitude of the value reported for that of *E. coli* K-12 [36].

References

1. Keefer, L.K.; Nims, R.W.; Davies, K.M.; Wink, D.A. "NONOates" (1-substituted diazen-1-ium-1,2-diolates) as nitric oxide donors: Convenient nitric oxide dosage forms. *Methods Enzymol.* **1996**, *268*, 281–293.
2. Lewis, R.S.; Deen, W.M. Kinetics of the reaction of nitric oxide with oxygen in aqueous solutions. *Chem. Res. Toxicol.* **1994**, *7*, 568–574.
3. Bolgiano, B.; Salmon, I.; Poole, R.K. Reactions of the membrane-bound cytochrome *bo* terminal oxidase of *Escherichia coli* with carbon monoxide and oxygen. *Biochim. Biophys. Acta* **1993**, *1141*, 95–104.
4. Mason, M.G.; Shepherd, M.; Nicholls, P.; Dobbin, P.S.; Dodsworth, K.S.; Poole, R.K.; Cooper, C.E. Cytochrome *bd* confers nitric oxide resistance to *Escherichia coli*. *Nat. Chem. Biol.* **2009**, *5*, 94–96.
5. Rice, C.W.; Hempfling, W.P. Oxygen-limited continuous culture and respiratory energy conservation in *Escherichia coli*. *J. Bacteriol.* **1978**, *134*, 115–124.
6. Junemann, S.; Butterworth, P.J.; Wrigglesworth, J.M. A suggested mechanism for the catalytic cycle of cytochrome *bd* terminal oxidase based on kinetic analysis. *Biochemistry* **1995**, *34*, 14861–14867.
7. Leif, H.; Sled, V.D.; Ohnishi, T.; Weiss, H.; Friedrich, T. Isolation and characterization of the proton-translocating NADH: Ubiquinone oxidoreductase from *Escherichia coli*. *Eur. J. Biochem.* **1995**, *230*, 538–548.
8. Verkhovskaya, M.L.; Belevich, N.; Euro, L.; Wikstrom, M.; Verkhovsky, M.I. Real-time electron transfer in respiratory complex I. *Proc. Natl. Acad. Sci. USA* **2008**, *105*, 3763–3767.
9. David, P.; Baumann, M.; Wikstrom, M.; Finel, M. Interaction of purified NDH-1 from *Escherichia coli* with ubiquinone analogues. *Biochim. Biophys. Acta* **2002**, *1553*, 268–278.
10. Jaworowski, A.; Campbell, H.D.; Poulis, M.I.; Young, I.G. Genetic identification and purification of the respiratory NADH dehydrogenase of *Escherichia coli*. *Biochemistry* **1981**, *20*, 2041–2047.
11. Villegas, J.M.; Volentini, S.I.; Rintoul, M.R.; Rapisarda, V.A. Amphipathic C-terminal region of *Escherichia coli* NADH dehydrogenase-2 mediates membrane localization. *Arch. Biochem. Biophys.* **2011**, *505*, 155–159.
12. Cotter, P.A.; Chepuri, V.; Gennis, R.B.; Gunsalus, R.P. Cytochrome *o* (*cyoABCDE*) and *d* (*cydAB*) oxidase gene expression in *Escherichia coli* is regulated by oxygen, pH, and the *fnr* gene product. *J. Bacteriol.* **1990**, *172*, 6333–6338.
13. Bekker, M.; Kramer, G.; Hartog, A.F.; Wagner, M.J.; de Koster, C.G.; Hellingwerf, K.J.; de Mattos, M.J. Changes in the redox state and composition of the quinone pool of *Escherichia coli* during aerobic batch-culture growth. *Microbiology* **2007**, *153*, 1974–1980.
14. Sundararaj, S.; Guo, A.; Habibi-Nazhad, B.; Rouani, M.; Stothard, P.; Ellison, M.; Wishart, D.S. The CyberCell Database (CCDB): A comprehensive, self-updating, relational database to coordinate and facilitate in silico modeling of *Escherichia coli*. *Nucleic Acids Res.* **2004**, *32*, D293–D295.
15. Taniguchi, Y.; Choi, P.J.; Li, G.W.; Chen, H.; Babu, M.; Hearn, J.; Emili, A.; Xie, X.S. Quantifying *E. coli* proteome and transcriptome with single-molecule sensitivity in single cells. *Science* **2010**, *329*, 533–538.
16. Volkmer, B.; Heinemann, M. Condition-dependent cell volume and concentration of *Escherichia coli* to facilitate data conversion for systems biology modeling. *PLoS ONE* **2011**, *6*, e23126.
17. Gardner, A.M.; Martin, L.A.; Gardner, P.R.; Dou, Y.; Olson, J.S. Steady-state and transient kinetics of *Escherichia coli* nitric-oxide dioxygenase (flavohemoglobin)—The B10 tyrosine hydroxyl is essential for dioxygen binding and catalysis. *J. Biol. Chem.* **2000**, *275*, 12581–12589.
18. Kim, S.O.; Orii, Y.; Lloyd, D.; Hughes, M.N.; Poole, R.K. Anoxic function for the *Escherichia coli* flavohaemoglobin (Hmp): reversible binding of nitric oxide and reduction to nitrous oxide. *FEBS Lett.* **1999**, *445*, 389–394.
19. Kennell, D.; Riezman, H. Transcription and translation initiation frequencies of the *Escherichia coli lac* operon. *J. Mol. Biol.* **1977**, *114*, 1–21.
20. Liang, S.; Bipatnath, M.; Xu, Y.; Chen, S.; Dennis, P.; Ehrenberg, M.; Bremer, H. Activities of constitutive promoters in *Escherichia coli*. *J. Mol. Biol.* **1999**, *292*, 19–37.
21. So, L.H.; Ghosh, A.; Zong, C.; Sepulveda, L.A.; Segev, R.; Golding, I. General properties of transcriptional time series in *Escherichia coli*. *Nat. Genet.* **2011**, *43*, 554–560.
22. Allen, R.C.; Popat, R.; Diggle, S.P.; Brown, S.P. Targeting virulence: can we make evolution-proof drugs? *Nat. Rev. Microbiol.* **2014**, *12*, 300–308.
23. Wang, C.; Trudel, L.J.; Wogan, G.N.; Deen, W.M. Thresholds of nitric oxide-mediated toxicity in human lymphoblastoid cells. *Chem. Res. Toxicol.* **2003**, *16*, 1004–1013.

24. Bernstein, J.A.; Khodursky, A.B.; Lin, P.H.; Lin-Chao, S.; Cohen, S.N. Global analysis of mRNA decay and abundance in *Escherichia coli* at single-gene resolution using two-color fluorescent DNA microarrays. *Proc. Natl. Acad. Sci. USA* **2002**, *99*, 9697–9702.
25. Selinger, D.W.; Saxena, R.M.; Cheung, K.J.; Church, G.M.; Rosenow, C. Global RNA half-life analysis in *Escherichia coli* reveals positional patterns of transcript degradation. *Genome Res.* **2003**, *13*, 216–223.
26. Bieling, P.; Beringer, M.; Adio, S.; Rodnina, M.V. Peptide bond formation does not involve acid-base catalysis by ribosomal residues. *Nat. Struct. Mol. Biol.* **2006**, *13*, 423–428.
27. Johansson, M.; Bouakaz, E.; Lovmar, M.; Ehrenberg, M. The kinetics of ribosomal peptidyl transfer revisited. *Mol. Cell* **2008**, *30*, 589–598.
28. Liang, S.T.; Xu, Y.C.; Dennis, P.; Bremer, H. mRNA composition and control of bacterial gene expression. *J. Bacteriol.* **2000**, *182*, 3037–3044.
29. Mosteller, R.D.; Goldstein, R.V.; Nishimoto, K.R. Metabolism of Individual Proteins in Exponentially Growing *Escherichia coli*. *J. Biol. Chem.* **1980**, *255*, 2524–2532.
30. Willetts, N.S. Intracellular protein breakdown in growing cells of *Escherichia coli*. *Biochem. J.* **1967**, *103*, 462–466.
31. Gardner, A.M.; Helmick, R.A.; Gardner, P.R. Flavorubredoxin, an inducible catalyst for nitric oxide reduction and detoxification in *Escherichia coli*. *J. Biol. Chem.* **2002**, *277*, 8172–8177.
32. Vicente, J.B.; Scandurra, F.M.; Forte, E.; Brunori, M.; Sarti, P.; Teixeira, M.; Giuffre, A. Kinetic characterization of the *Escherichia coli* nitric oxide reductase flavorubredoxin. *Methods Enzymol.* **2008**, *437*, 47–62.
33. Gardner, A.M.; Gardner, P.R. Flavohemoglobin detoxifies nitric oxide in aerobic, but not anaerobic, *Escherichia coli*—Evidence for a novel inducible anaerobic nitric oxide-scavenging activity. *J. Biol. Chem.* **2002**, *277*, 8166–8171.
34. Silaghi-Dumitrescu, R.; Ng, K.Y.; Viswanathan, R.; Kurtz, D.M., Jr. A flavo-diiron protein from *Desulfovibrio vulgaris* with oxidase and nitric oxide reductase activities. Evidence for an in vivo nitric oxide scavenging function. *Biochemistry* **2005**, *44*, 3572–3579.
35. Poole, R.K.; Anjum, M.F.; MembrilloHernandez, J.; Kim, S.O.; Hughes, M.N.; Stewart, V. Nitric oxide, nitrite, and fnr regulation of *hmp* (Flavohemoglobin) gene expression in *Escherichia coli* K-12. *J. Bacteriol.* **1996**, *178*, 5487–5492.
36. Bennett, B.D.; Kimball, E.H.; Gao, M.; Osterhout, R.; Van Dien, S.J.; Rabinowitz, J.D. Absolute metabolite concentrations and implied enzyme active site occupancy in *Escherichia coli*. *Nat. Chem. Biol.* **2009**, *5*, 593–599.
37. Keseler, I.M.; Mackie, A.; Peralta-Gil, M.; Santos-Zavaleta, A.; Gama-Castro, S.; Bonavides-Martinez, C.; Fulcher, C.; Huerta, A.M.; Kothari, A.; Krummenacker, M.; *et al.* EcoCyc: fusing model organism databases with systems biology. *Nucleic Acids Res.* **2013**, *41*, D605–D612.

© 2016 by the authors; licensee MDPI, Basel, Switzerland. This article is an open access article distributed under the terms and conditions of the Creative Commons by Attribution (CC-BY) license (http://creativecommons.org/licenses/by/4.0/).
